# Pyrazolo Derivatives as Potent Adenosine Receptor Antagonists: An Overview on the Structure-Activity Relationships

**DOI:** 10.1155/2011/480652

**Published:** 2011-03-07

**Authors:** Siew Lee Cheong, Gopalakrishnan Venkatesan, Priyankar Paira, Ramasamy Jothibasu, Alexander Laurence Mandel, Stephanie Federico, Giampiero Spalluto, Giorgia Pastorin

**Affiliations:** ^1^Department of Pharmacy, National University of Singapore, 3 Science Drive 2, Singapore 117543; ^2^Dipartimento di Scienze Farmaceutiche, Università degli Studi di Trieste, Piazzale Europa 1, 34127 Trieste, Italy

## Abstract

In the past few decades, medicinal chemistry research towards potent and selective antagonists of human adenosine receptors (namely, A_1_, A_2A_, A_2B_, and A_3_) has been evolving rapidly. These antagonists are deemed therapeutically beneficial in several pathological conditions including neurological and renal disorders, cancer, inflammation, and glaucoma. Up to this point, many classes of compounds have been successfully synthesized and identified as potent human adenosine receptor antagonists. In this paper, an overview of the structure-activity relationship (SAR) profiles of promising nonxanthine pyrazolo derivatives is reported and discussed. We have emphasized the SAR for some representative structures such as pyrazolo-[4,3-*e*]-1,2,4-triazolo-[1,5-*c*]pyrimidines; pyrazolo-[3,4-*c*] or -[4,3-*c*]quinolines; pyrazolo-[4,3-*d*]pyrimidinones; pyrazolo-[3,4-*d*]pyrimidines and pyrazolo-[1,5-*a*]pyridines. This overview not only clarifies the structural requirements deemed essential for affinity towards individual adenosine receptor subtypes, but it also sheds light on the rational design and optimization of existing structural templates to allow us to conceive new, more potent adenosine receptor antagonists.

## 1. Introduction

Adenosine is an endogenous nucleoside that mediates a wide range of physiological responses through interaction with specific adenosine receptors (ARs), which are G-protein-coupled receptors (GPCRs) comprising the characteristic seven transmembrane domains connected by three extracellular and three intracellular loops. There are four basic types of ARs that have been cloned and pharmacologically characterized, namely, A_1_, A_2A_, A_2B_, and A_3_ ARs [1]. Each of these ARs is associated with its own distinct biochemical pathways. Typically, the activation of A_1_ and A_3_ receptors mediates adenylyl cyclase inhibition through an interaction with G_i_ protein, followed by a subsequent decrease in the level of cyclic adenosine monophosphate (cAMP); conversely, the A_2A_ and A_2B_ receptors stimulate the adenylyl cyclase activity via the G_s_ protein thereby increasing the level of cAMP [2]. In addition, other signaling pathways involving phospholipases C and D, and Ca^2+^ and mitogen-activated protein kinases (MAPK) have also been described [1]. Pharmacologically, the inhibition of A_1_ receptors has led to implications in the renal system disorders through regulation of diuresis and neurological disorders such as Alzheimer's disease [3, 4]; on the other hand, A_3_ receptor antagonists are primarily related to the treatment of glaucoma, renal protection, inflammatory disorders like asthma, as well as cancer [5–7]. Studies have also found that A_2A_ receptor antagonists can reverse Parkinsonian motor deficits in preclinical models of Parkinson's disease, and they do so without inducing or exacerbating dyskinesias in nonhuman primate models [8, 9]. As for the A_2B_ receptor, its antagonists seem to be suitable for the treatment of certain forms of inflammatory processes such as asthma via modulation of mast cell degranulation [10, 11]. 

 In the last 15 years, intensive efforts in medicinal chemistry to design and synthesize new AR antagonists have led to the discovery of potent and selective ligands (with either agonistic or antagonistic properties) for the A_1_, A_2A_, A_2B_, and A_3_ ARs. These new derivatives have resulted in a better understanding of the pathophysiological role of these receptors; more precisely, among the AR antagonists, several different types of xanthine-derived and nonxanthine-based polyheterocyclic structures have been identified as potent AR antagonists. Some of them are shown to possess good affinity exclusively towards a particular AR subtype with concomitant improvements in their selectivity profiles. On the other hand, some scaffolds demonstrate good binding affinity across more than one AR subtype, with relatively lower selectivity profile. Among these diverse classes of compounds, nonxanthine pyrazolo derivatives have been reported to show good potency towards ARs, together with a broad range of selectivity. The aim of this review is to briefly summarize the structure-activity relationship profiles of various nonxanthine derivatives containing the pyrazole moiety as AR antagonists to the A_1_, A_2A_, A_2B_, and A_3_ receptor subtypes.

## 2. Pyrazolo Derivatives as Potent AR Antagonists

In general, nonxanthine AR antagonists are represented by polyheterocyclic derivatives which are categorized as monocyclic, bicyclic, or tricyclic structures [12]. In this review, we emphasized the structure-activity relationships for some of the representative nonxanthine pyrazolo derivatives (i.e., derivatives with a fused pyrazole ring in their respective core nuclei), which have been identified as potent AR antagonists at the A_1_, A_2A_, A_2B_, or A_3_ receptor subtypes. These derivatives are pyrazolo-[4,3-*e*]1,2,4-triazolo-[1,5-*c*]pyrimidines, pyrazolo-[3,4-*c*] or -[4,3-*c*] quinolines, pyrazolo-[4,3-*d*]pyrimidinones, pyrazolo-[3,4-*d*]pyrimidines, and pyrazolo-[1,5-*a*]pyridines. The binding data of the most representative AR antagonists belonging to these series are reported in Table 1.

### 2.1. Pyrazolo-[4,3-*e*]-1,2,4-triazolo-[1,5-*c*]pyrimidine

#### 2.1.1. A_2A_ AR Antagonists

**Figure 1:**
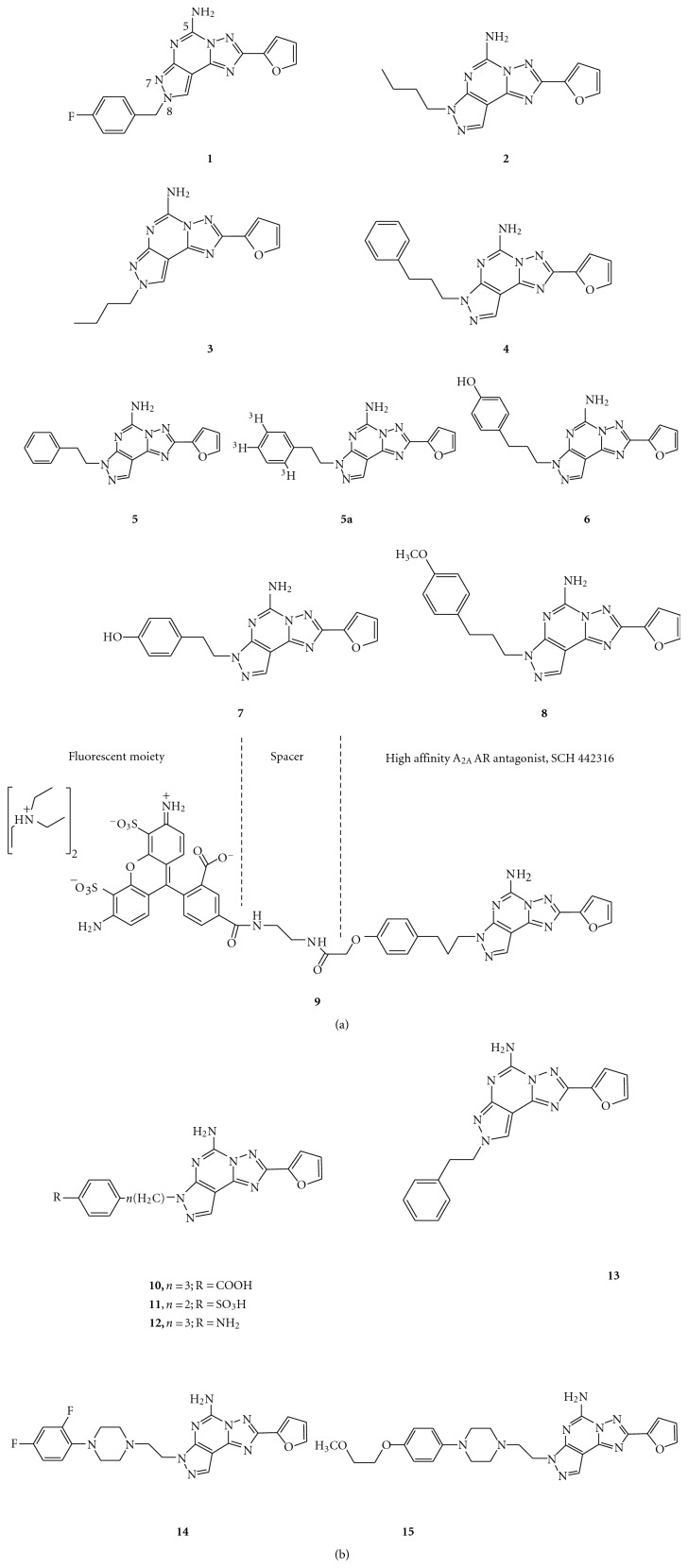
Structures of pyrazolo-triazolo-pyrimidines as A_2A_ AR antagonists.

The pyrazolo-triazolo-pyrimidine derivatives were first described as AR antagonists by Gatta and coworkers [13], who identified a compound named 8FB-PTP (**1** in Figure 1), which demonstrated good binding to the A_2A_ AR but lacked selectivity towards the A_1_ receptor subtype. Structure-affinity relationship studies showed that the free amino group at 5-position and the effect of the substituents on the pyrazole ring seemed important for both high affinity and selectivity for the A_2A_ AR subtype. From further studies, substitutions at the 7-position were shown to improve the selectivity for the A_2A_ receptor while the same substitutions at the 8-position increased affinity to the A_1_ and A_2A_ receptors with low levels of selectivity, as indicated by the N^7^-*n*-butyl (**2**) and the N^8^-*n*-butyl (**3**) derivatives [14, 15]. This again indicated that the presence of a chain (preferably a long (ar)alkyl one) at the N^7^ position seemed essential for both affinity and selectivity for the A_2A_ receptors. 

 In fact, two selected compounds named SCH 63390 (**4**) and SCH 58261 (**5**) proved to be the most potent and selective A_2A_ AR antagonists ever reported, both in rat and human models [15–17]. The latter was further developed into an A_2A_ antagonist radioligand, [^3^H]SCH 58261 (**5a**) with a *K*_*D*_ value of about 1 nM. Further studies have suggested that it could be a useful tool for characterization of A_2A_ receptor subtypes in platelets, autoradiography assays, and labeling of striatal A_2A_ receptors for studying A_2A_ receptor occupancy of various antagonists [18, 50, 51]. Nevertheless, this class of compounds presents a significant problem because of poor water solubility. To overcome this drawback, several polar moieties on the side chain of the pyrazole nucleus have been introduced. In particular, the introduction of a hydroxyl function at the *para* position of the phenyl ring of compounds (**4**) and (**5**) led to derivatives (5-amino-7-[3-(4-hydroxyphenyl)propyl]-2-(2-furyl)pyrazolo[4,3-*e*]1,2,4-triazolo[1,5-*c*]pyrimidine) (**6**) and (5-amino-7-[*β*-(4-hydroxyphenyl)ethyl]-2-(2-furyl)pyrazolo[4,3-*e*]1,2,4-triazolo[1,5-c]pyrimidine) (**7**), which not only showed a better hydrophilic character but also a significant increase of both affinity and selectivity for the A_2A_ AR subtype, suggesting that most probably, a hydrogen bond is involved in receptor recognition via this part of the ligand [16]. 

 To understand the nature of such a hypothetical hydrogen bond, compound SCH 442416 (**8**) was synthesized. This derivative showed even higher affinity and selectivity for the A_2A_ receptor, thus representing a suitable candidate for positron emission tomography (PET) studies in its ^11^C-labeled form [19]. Moreover, it was developed into novel fluorescent tracer MRS5346 (**9**), which was conjugated to the fluorescent dye Alexa Fluor-488. It has a *K*_*D*_ value of 16.5 ± 4.7 nM and could be used in fluorescence polarization competition binding experiments as well as high-throughput screening [20]. On top of that, this SCH 442416 derivative also confirmed the role of a hydrogen bond via the pyrazolo side chain. Nonetheless, the introduction of oxygenated groups could not be considered sufficient to confer water solubility. Hence, carboxylic (**10**) and sulfonic (**11**) moieties were introduced, and such structural modifications (the sulfonic moiety in particular) improved water solubility. However, in some cases, a loss of affinity with respect to reference compounds (**6**,** 7**) for the A_2A_ AR was observed. On the other hand, the introduction of an amino group at the *para* position of the phenyl ring (**12**) improved both affinity and selectivity towards the A_2A_receptor, although with low water solubility [17]. Despite these observations, it was found that the N^7^ derivative (such as compound **5**) was totally inactive to the human A_2B_ and A_3_ receptors. The N^8^ regioisomer (**13**), however, showed a slight affinity profile for these two receptor subtypes [21, 22]. 

A recent series of pyrazolo-triazolo-pyrimidine derivatives was obtained by modifying the phenylethyl substituent of **5 **with substituted phenylpiperazine ethyl groups [23]. Introduction of fluorine atoms in the phenyl ring (**14**) enhanced the affinity to subnanomolar values and the compounds displayed potent oral activity, but their solubilities still remained poor. Further introduction of ether substituents led to derivatives with high affinities and selectivities for A_2A_ receptors and improvements in water solubility. In particular, one of these compounds (SCH 420814, Privadenant, **15**) exhibited high affinity for both rat and human A_2A_ receptors, with *K*_*i*_ values of 2.5 and 1.1 nM, respectively. In addition, the compound was very selective for human A_2A_ receptors over A_1_, A_2B_, and A_3_ receptors. Interestingly, the compound did not show significant binding against a panel of 59 unrelated receptors, enzymes and ion channels. Privadenant is now in Phase II Clinical Trials for the treatment of dyskinesia in Parkinson's disease.

#### 2.1.2. A_2B_ AR Antagonists

**Figure 2:**
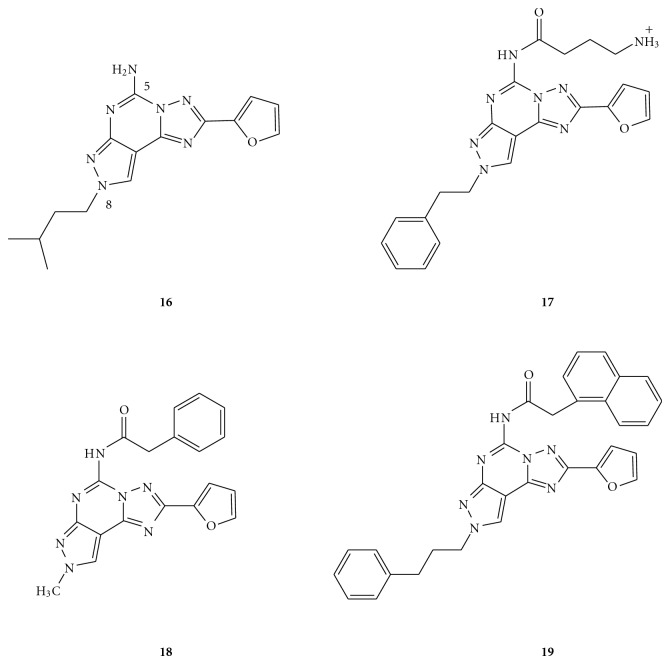
Structures of pyrazolo-triazolo-pyrimidines as A_2B_ AR antagonists.

The binding data obtained from parallel studies on A_2A_ receptor antagonists have indicated that the N^5^-unsubstituted pyrazolo-triazolo-pyrimidine derivatives (**13 **in Figure 1, **16 **in Figure 2) possessed high affinity to the human A_2B_ receptors but completely lacked selectivity. Subsequently, introduction of a polar *γ*-amino-butyryl amide (**17**) at the N^5^-position decreased affinity towards the A_2B_ receptors but was found to be slightly selective against the A_2A_ subtype [24]. An improvement of this class of compounds was further achieved by an optimized pattern of substitutions at the N^5^- and N^8^-positions. In fact, in parallel studies on human A_3_ receptor antagonists (to be elaborated in the following section), it was observed that replacement of the phenylcarbamoyl moiety at the N^5^-position with a phenylacetyl group (compound **18**) produced a decrease in affinity to the human A_3_ AR and a retention or improvement towards the A_2B_ subtype. A combination of a naphthyl acetyl moiety at the N^5^-position and a phenyl propyl group (characteristic of A_2A_ antagonists) at the N^8^ position led to a compound (**19**), which was found to be quite potent and selective towards the A_2B_ ARs [25]. These findings indicated that bulky substituents at both the N^5^- and N^8^-positions could lead to potent and selective A_2B_ AR antagonists, thus suggesting the presence of a larger pocket in the receptor binding site.

#### 2.1.3. A_3_ AR Antagonists

**Figure 3:**
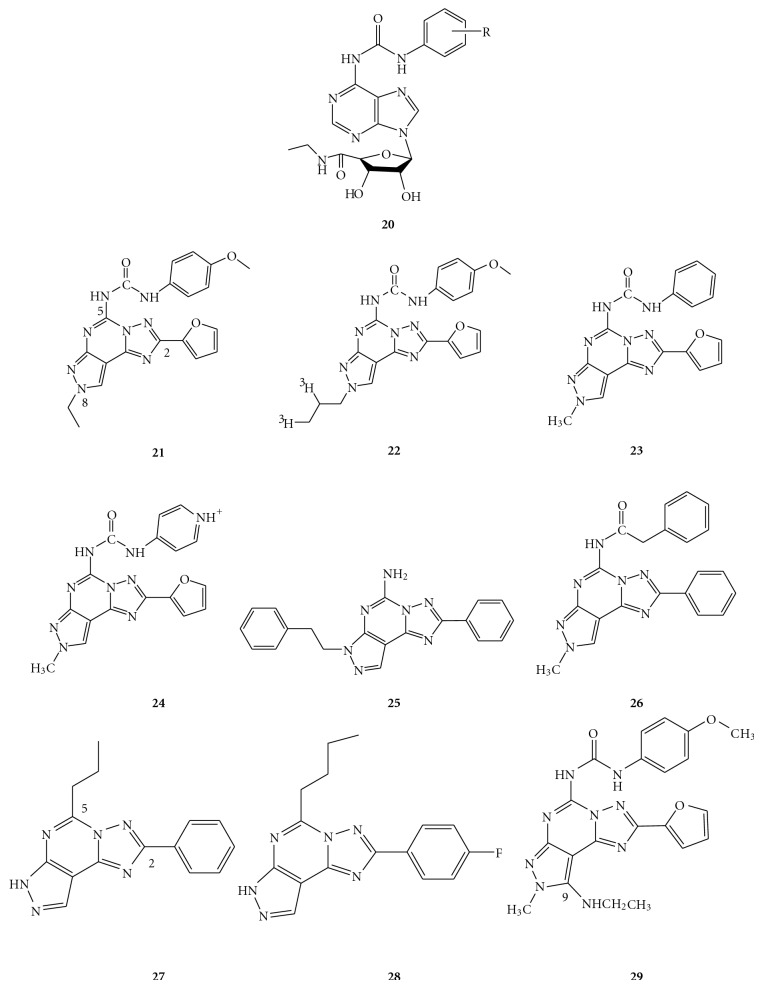
Structures of (a) N^6^-(substituted arylcarbamoyl) adenosine-5′-uronamide as A_3_ AR agonist; (b) pyrazolo-triazolo-pyrimidines as A_3_ AR antagonists.

The optimization approach to obtain potent A_3_ AR antagonists in the series of pyrazolo-triazolo-pyrimidines was a hybrid molecule between a human A_2A_ receptor antagonist [15, 16] and an agonist of the A_3_ subtype [52, 53]. The tricyclic scaffold of a known human A_2A_ antagonist was substituted at the N^5^ position with an aryl carbamoyl moiety. Specifically, this *para*-methoxyphenyl was demonstrated to be optimal for A_3_ affinity when introduced at the N^6^-position of the A_3_ agonist NECA (as represented by compound **20**; Figure 3). Such rational design led to compound **21**, which is one of the most potent and selective human A_3_ AR antagonists [21]. Subsequent collation of binding data and molecular modeling studies indicated that small substituents, such as a methyl group at the N^8^-position, the phenyl ring on the N^5^-carbamoyl moiety, and a furyl ring at 2-position, were important (although not crucial, as indicated in the following paragraphs) for A_3_ affinity (e.g., compound **23**) [22, 27–29]. Only small substituents at the *para* position of the phenyl ring, including fluoro (F), chloro (Cl), and methoxy (OCH_3_) were tolerated. At the *meta*-position, only hydrogen was tolerated, while the *ortho*-position could be substituted by a chlorine atom. Introduction of an allyl chain at N^8^-position, followed by reduction with tritium afforded [^3^H]MRE-3008-F20 (**22**), which was the first selective and tritiated human A_3_ receptor antagonist radioligand [26]. It showed a *K*_*D*_ value of 0.8 nM and exhibited ca. 25% of nonspecific binding at that concentration. Since its discovery, it has been used for the identification of A_3_ receptors on various cells, including Jurkat T cells, HL60 cells, and human neutrophils [54, 55]. Later, the N^5^-phenyl ring of the tricyclic scaffold was substituted with a pyridinium salt, as represented by compound **24**, which not only showed good solubility (15 mM) but also significantly improved hA_3_ affinity [30]. In previous studies, substitution of the N^5^-pyridine moiety with various N^5^-heteroaryl rings resulted in a general loss of hA_3_ affinity and selectivity [28]. 

 Substitution at position C^2^ of the tricyclic system has not been deeply explored, being essentially limited to a furyl group. The furan ring had been considered to be an essential structural requirement for the binding of antagonists to all of the AR subtypes, since its removal from the tricyclic system was associated with an irreversible loss of affinity and selectivity, regardless of the receptor under investigation. In fact, Baraldi and coworkers [31] found that the substitution of the furan ring in PTPs with phenyl (**25**) or alkoxyphenyl rings led to a loss of affinity to A_2A_, A_2B_, and A_3_ receptors, while the A_1_ subtype in some cases displayed a high nanomolar binding profile. Similarly, the functionalization of the furan ring with polar substituents led to completely inactive derivatives, clearly indicating that an unsubstituted furan ring at the C^2^ position played a fundamental role in ligand-receptor recognition [31]. Notably, in most cases, substitution at the pyrazole ring occurred at the N^7^-rather than at the N^8^-position. Recently, a new series of 2-aryl pyrazolo-triazolo-pyrimidines was reported by Cheong et al., in which the previously conserved furan at C^2^ was substituted with a 2-aryl ring while substitutions on pyrazole ring were maintained at the N^8^-position [32]. Such bioisosteric replacement at C^2^ resulted in improved human A_3_ affinity and remarkably enhanced selectivity over other AR subtypes. The *para* substituents at the 2-phenyl ring were generally well tolerated, except for a *para*-nitro group, which caused detrimental effects on hA_3_ affinity. Particularly, the *para*-OCH_3_ and *para*-F groups conferred better affinities and selectivities towards the hA_3_ receptor. The most potent compound in this series (**26**) had a methyl group at the N^8^-position, a phenylacetamide at the N^5^-position, and a phenyl ring at the C^2^-position. Interestingly, Okamura et al. also described a series of pyrazolo-triazolo-pyrimidine analogues with a *para*-(un)substituted-phenyl ring and an alkyl chain at the C^2^- and C^5^-positions, respectively, that was shown to possess good hA_3_ affinity. The selectivity against other AR subtypes was significantly improved in this group of derivatives, especially when a *para*-substituted-2-phenyl ring was present (as illustrated by compounds **27**,** 28**) [6, 33]. It was also observed that the introduction of a substituent (e.g., NHCH_2_CH_3_ (**29**) and SCH_3_) at the C^9^-position, induced a loss of both affinity and selectivity towards the A_3_ receptor. It was postulated that the introduction of these substituents caused a repulsive effect due to steric hindrance, which hampered the interaction with the binding site of the A_3_ AR [31].

### 2.2. Pyrazolo-[3,4-*c*] or -[4,3-*c*]quinolines

#### 2.2.1. A_3_ AR Antagonists

**Figure 4:**
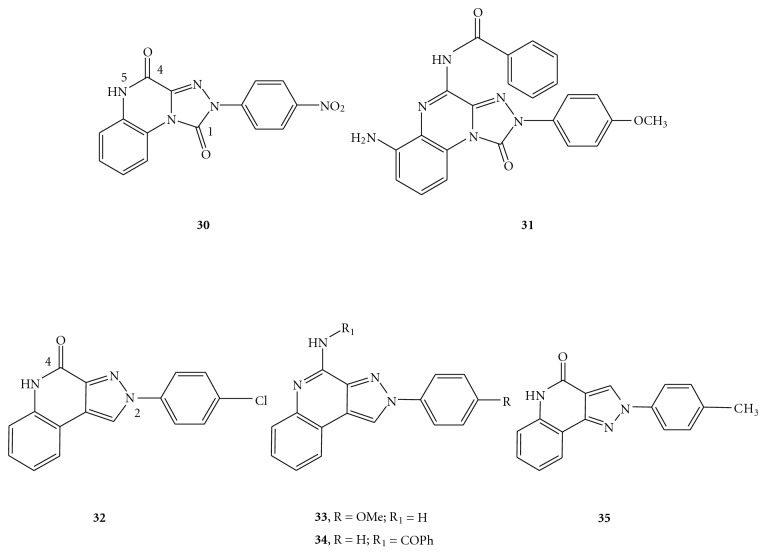
Structures of pyrazoloquinolines as A_3_ AR antagonists.

The series of pyrazoloquinolin-4-ones and pyrazolo[3,4-*c*]quinolines, 4-oxo and 4-amino substituted, shared a similar central scaffold as that of the triazoloquinoxalinones (**30**,** 31**) [34–38], and they were found to be potent and selective A_3_ AR antagonists (Figure 4) [39, 40]. The substituent on the appended 2-phenyl ring was crucial to modulate A_3_ affinity while a nuclear (e.g., oxo group) or extranuclear (e.g., amide group) C=O proton acceptor at the 4-position gave rise to potent and selective A_3_ antagonists. At the 2-position, the presence of 4-Cl, 4-OCH_3_, 4-CH_3_, and 3-CH_3_ on the 2-phenyl ring resulted in enhancement of A_3_ affinities in both the 4-ones (**32**) and 4-amino (**33**) series. Conversely, the substituents on the 2-phenyl ring of the 4-amido derivatives generally maintained but did not ameliorate the high A_3_ affinities in comparison with the 2-phenyl parent derivatives. At the 4-position, the introduction of 4-benzoylamido (**34**), 4-phenylacetylamido, and 4-carbamoyl residues resulted in improved human A_3_ affinities and selectivities, confirming the importance of the C=O group at this position towards A_3_ receptor-ligand interaction. Among the 4-amido derivatives, the 4-acetylamido group showed lower human A_3_ affinity in comparison to the other bulkier 4-acyl moieties, thus implying not only the presence of a roomy receptor pocket around this region, but also the importance of hydrophobic interactions between the 4-substituents and the receptor site. Another series of 2-phenyl-2,5-dihydro-pyrazolo[4,3-*c*]quinolin-4-ones, which are the structural isomers of the parent 2-arylpyrazolo[3,4-*c*]quinoline derivatives, have also been reported by Baraldi et al. [41]. Some of the synthesized compounds showed good A_3_ affinities (nanomolar ranges) and excellent selectivities. Particularly, the substitution of methyl, methoxy, or chlorine at the *para*-position of the 2-phenyl ring, together with the presence of a 4-oxo functionality gave good A_3_ affinity and selectivity (**35**).

### 2.3. Pyrazolo-[4,3-*d*]pyrimidinones

#### 2.3.1. A_3_ AR Antagonists

**Figure 5:**
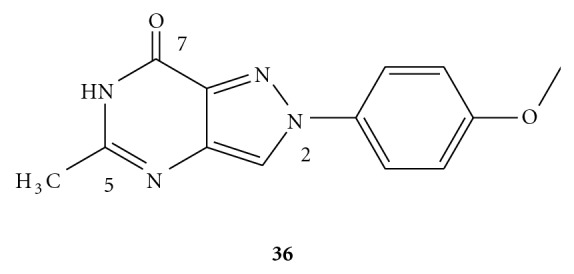
Structure of pyrazolo-[4,3-*d*]pyrimidinone as an A_3_ AR antagonist.

The pyrazolo-[4,3-*d*]pyrimidin-7-ones, which were a molecular simplification of the tricyclic scaffold of pyrazolo-[3,4-*c*]quinolin-4-one, have recently been shown to possess good affinity and selectivity profiles for the hA_3_ receptor [42]. According to the structure-activity relationship (SAR) analysis, both the substituents at the C^5^- and N^2^-positions of the bicyclic nucleus were crucial for the human A_3_ affinity and selectivity. The concomitant presence of small alkyl chains, such as methyl group at the C^5^-position and a *para*-methoxy-substituted phenyl ring at the N^2^ position (as demonstrated by compound **36** in Figure 5) gave rise to the most potent and selective A_3_ AR antagonist in this series of derivatives.

### 2.4. Pyrazolo-[3,4-*d*]pyrimidines

#### 2.4.1. A_1_ AR Antagonists

**Figure 6:**
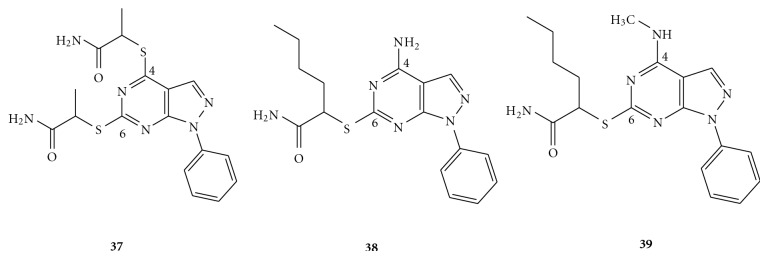
Structures of pyrazolo-[3,4-*d*]pyrimidines as A_1_ AR antagonists.

A series of pyrazolo-[3,4-*d*]pyrimidines was identified that contains novel A_1_ AR antagonists [43]. The lead compound, 4,6-Bis[*α*-carbamoylethyl)thio]-1-phenylpyrazolo-[3,4-*d*]pyrimidine (**37** in Figure 6), served as a starting template for the optimization of A_1_ affinity and selectivity in this series of compounds. 1-phenyl-pyrazolo-[3,4-*d*]pyrimidine was modified at C^4^ with mercapto, methylthio, and amino groups in order to investigate the hydrogen-bonding and steric tolerance at this position [44]. At C^6^, thioesters containing distal amides with varying lengths of linear and branched alkyl groups extending from the *α*-carbon were evaluated for steric and hydrophobic tolerance [44]. From the binding data at A_1_ receptor, it was found that the simultaneous presence of an amino at C^4^ and *α*-butyl side chain at C^6^ gave rise to the most potent compound of the series (**38**); the least potent compound contained a mercapto and an *α*-isopropyl side chain at C^4^ and C^6^, respectively. These observations suggested that the superiority of the C^4^-amino group was most likely due to a hydrogen-bonding interaction with the receptor binding sites. Although a C^4^-methylthio group was less preferable than the amino species, its presence was still tolerable, thus indicating the existence of a hydrophobic pocket in the A_1_ binding site able to accommodate the methyl group. As for the C^6^ position, the increase in length of the linear carbon side chain (from ethyl to butyl) was favorably tolerated at the A_1_ receptor for each C^4^-substituent. Similarly, the hydrophobic tolerance at C^6^ position seemed crucial for the A_1_ binding affinity as well. In an attempt to test for the hypothesis mentioned above, a methyl-amino and an *α*-butyl side chain were concurrently introduced at the C^4^ and C^6^ positions, respectively [44]. Accordingly, the derivative **39** displayed improved A_1_ affinity and increased A_1_ selectivity, which further supported the proposed structural requirements at both the C^4^ and C^6^ positions.

#### 2.4.2. A_2A_ AR Antagonists

**Figure 7:**
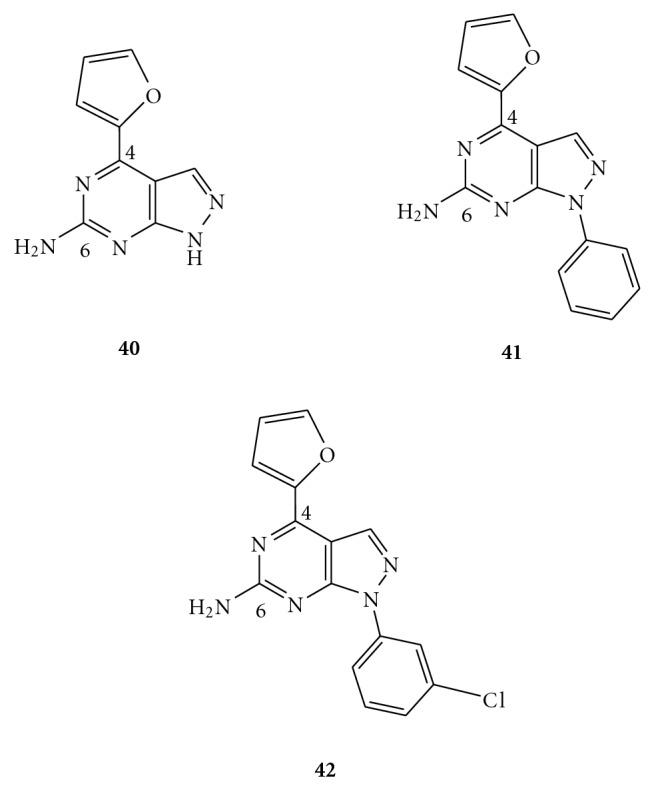
Structures of pyrazolo-[3,4-*d*]pyrimidines as A_2A_ AR antagonists.

Pyrazolo-[3,4-*d*]pyrimidines were also explored by Gillespie and collaborators as A_2A_ AR antagonists [45]. In particular, the 4-(furan-2-yl)pyrazolo-[3,4-*d*]pyrimidine (compound **40** in Figure 7) was identified as a starting point for further investigation. It showed a good affinity for the A_2A_ receptor subtype and was 13-fold more selective over A_1_. The following introduction of 1-phenyl substitution (**41**) increased potency at A_2A_ while either incorporation of heteroatoms or ring saturation did not improve affinity significantly. Extension of spatial linker between the phenyl ring and pyrazole by more than one methylene group was found to provide an hA_2A_ affinity profile similar to the 1-phenyl derivative. Furthermore, subsequent substitution on the *meta*-position of phenyl ring with electron-rich and deficient groups was tolerated, with the 3-chlorobenzyl derivative (**42**) demonstrating the best hA_2A_ affinity and selectivity in the series. Moreover, compounds **40–42** have also shown *in vivo* activity in a mouse haloperidol-induced hypolocomotion model of Parkinson's disease. Due to the fact that the 4-(furan-2-yl) moiety in this series of compounds could be easily converted into reactive species under oxidative metabolism, further studies were undertaken to replace such group with other nonfuran-containing heterocycles. Unfortunately, the resulting compounds have showed reduced affinity for the A_2A_ receptor.

#### 2.4.3. A_3_ AR Antagonists

**Figure 8:**
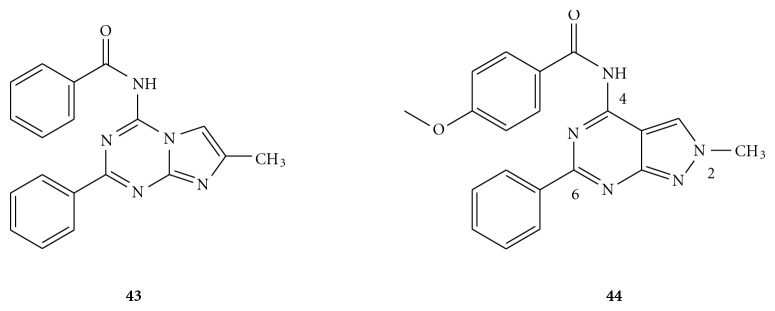
Structures of (a) parent scaffold, imidazole[1,2-*a*][1,3,5]triazine as an A_1_ and A_3_ AR antagonist; (b) pyrazolo-[3,4-*d*]pyrimidine as an A_3_ AR antagonist.

Pyrazolo-[3,4-*d*]pyrimidines represent a novel series of bicyclic scaffold-derived A_3_ antagonists [46] isosterically related to the imidazole-[1,2-*a*][1,3,5]triazine (**43**; Figure 8), which have shown a certain degree of binding affinity at both A_1 _and A_3_ receptors [56]. Such pyrazolo-pyrimidine analogues displayed improved A_3_ affinity and selectivity profiles in comparison to the parent imidazole-triazines. From the binding affinity results, it was suggested that the 6-phenyl substituent at the bicyclic scaffold was a key pharmacophoric element for recognition at the ARs, since its removal led to poor affinity to all the ARs. Besides that, small alkyl groups at the N^2^-position, such as a methyl moiety were found to be more favourable than bulky groups for conferring good human A_3_ affinity. The introduction of N^4^-acyl substituents generally resulted in improved human A_3_ affinity relative to unsubstituted derivatives. In particular, the presence of a methyl group at N^2^, together with para-methoxy benzoyl substituent at N^4^ (**44**) dramatically increased the potency and selectivity to the A_3_ AR. Compound **44** was subsequently tested on human glioma U87MG cells, and it was able to counteract the proliferation of glioma cells mediated by A_3_ AR agonists Cl-IB-MECA and IB-MECA through the inhibition of A_3_ AR agonist-mediated ERK 1/2 activation. This finding implied that this class of derivatives might represent promising lead compounds for the development of adjuvants for glioma chemotherapy [46].

### 2.5. Pyrazolo-[1,5-*a*]pyridines

#### 2.5.1. A_1_ AR Antagonists

**Figure 9:**
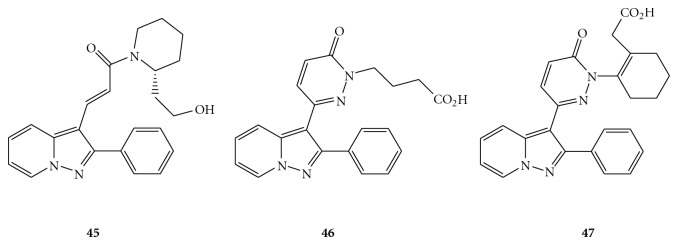
Structures of pyrazolo-[1,5-*a*]pyridine as A_1_ AR antagonists.

Akahane and coworkers reported a series of pyrazolo-[1,5-*a*]pyridine derivatives as potent and selective A_1_ AR antagonists. FK453 (**45 **in Figure 9) [47] and FK838 (**46**) [48] were the typical examples of such derivatives, and they also showed diuretic activity both *in vivo* and *in vitro*. Nevertheless, there were some limitations in these two compounds. For FK453, photochemical *trans-cis* isomerization at the acryloyl amide moiety and low water solubility (11.9 *μ*g/mL) were two main problems in this type of structure. In FK838, photochemical stability was achieved through the substitution of the acryloyl amide with a pyridazinone ring while water solubility (10 mg/mL) was enhanced by the introduction of the butyric acid group. Nevertheless, this derivative had lower binding affinity and poorer selectivity for A_1_ receptor than FK453. Subsequently, further structural modifications to FK838 led to the synthesis of FR166124 (**47**) [49], which is the most potent and selective A_1_ AR antagonist of this series, and it shows high water solubility (>200 mg/mL). In fact, it was designed based on the hypothesis that the high affinity and selectivity of FK453 for the A_1_ receptor was due to the presence of the (2*R*)-2-(2-hydroxyethyl)piperidine ring of the acryloyl amide as a conformationally limiting factor. The pyridazinone ring of FK838 was maintained in the structure of FR166124, with the introduction of a ring structure joining the C^3^ and C^4^ positions of the butyric acid group to limit possible conformations. Overall, the close resemblance of X-ray crystal structures of FR166124 and FK453 to each other, together with the experimental binding assay data, suggested that the presence of a double bond in the cyclohexenyl acetic acid group was essential for high selectivity to the A_1_ receptor, with good A_1_ affinity and water solubility.

## 3. Conclusion

Pyrazolo-containing polyheterocyclic scaffolds have given rise to a group of potent and selective antagonists for the A_1_, A_2A_, A_2B_, and A_3_ AR subtypes. An overview of the structure-activity relationships of each class of derivatives not only clarifies the structural requirements deemed essential for the affinity towards the individual AR subtypes, but it also lends insight into the rational design and optimization of existing structural templates to obtain other new, potent AR antagonists.

## Figures and Tables

**Table 1:** Binding affinities of pyrazolo derivatives at A_1_, A_2A_, A_2B_, and A_3_ ARs.

Pyrazolo derivatives						
Type of scaffold	Compounds	*K*_*i*_, affinity (nM) or % of inhibition				
		A_1_^a^	A_2A_^b^	A_2B_^c^	A_3_^d^	Refs.

Tricyclic scaffold	Pyrazolo-[4,3-*e*]triazolo-[1,5-*c*]pyrimidine					
	*A_2A_ AR antagonists*					
	**1**, 8FB-PTP	3.3^e^	1.2^f^	ND	ND	[13]
	**2**	236^e^	8.9^f^	ND	ND	[14, 15]
	**3**	30.4^e^	2.4^f^	ND	ND	[14, 15]
	**4**, SCH 63390	504^e^	2.4^f^	ND	>10,000	[15–17]
	**5**, SCH 58261	121^e^	2.3^f^	ND	>10,000	[15–17]
	**5a**, [^3^H]SCH 58261	—	*K*_*D*_ = 1 nM	—	—	[18]
	**6**	741^e^	0.94^f^	ND	>10,000	[16]
	**7**	444^e^	1.7^f^	ND	>10,000	[16]
	**8**, SCH 442416	1,111	0.048	>10,000	>10,000	[19]
	**9**, MRS5346	—	*K*_*D*_ = 16.5 nM	—	—	[20]
	**10**	4,927	4.63	>10,000	>10,000	[17]
	**11**	190	100	>10,000	>10,000	[17]
	**12**	2,160	0.22	>10,000	>10,000	[17]
	**13**	1	0.34	5.1	280	[21, 22]
	**14**	>960	0.6	ND	ND	[23]
	**15**, SCH 420814	>1,000	1.1	>1,700	>1,000	[23]
	*A_2B_ AR antagonists*					
	**16**	2	0.8	9	700	[24]
	**17**	1.6	54	27	65	[24]
	**18**	702	423	165	0.81	[25]
	**19**	1,100	800	20	300	[25]
	*A_3_ AR antagonists*					
	**21**	1,026	1,040	245	0.6	[21]
	**22,** [^3^H]MRE-3008-F20	—	—	—	*K*_*D*_ = 0.8 nM	[26]
	**23 **	594	381	222	0.16	[22, 27–29]
	**24**	350	100	250	0.01	[30]
	**25**	235	>1,000	>1,000	>1,000	[31]
	**26**	562	778	>10,000^g^	0.108	[32]
	**27**	38^h^	120^i^	1,500^j^	4.1^k^	[6, 33]
	**28**	610^h^	>10,000^i^	9,400^j^	1.9^k^	[6, 33]
	**29**	150	21	37	17	[31]
	Pyrazoloquinoline					
	*A_3_ AR antagonists*					
	**30**	32%	21%^m^	ND	0.6	[34–38]
	**31**	45%	24%	>1,000	1	[37]
	**32**	464^l^	35%^m^	ND	2.9	[39, 40]
	**33**	40	1,060	ND	90.2	[39, 40]
	**34**	0%	9%	ND	2.1	[39, 40]
	**35**	>1,000	>1,000	>1,000^n^	9.0	[41]
Bicyclic scaffold	Pyrazolo-[4,3-*d*]pyrimidinone					
	*A_3_ AR antagonist*					
	**36**	5%	1%	2%^g^	1.2	[42]
	Pyrazolo-[3,4-*d*]pyrimidine					
	*A_1_ AR antagonists*					
	**37**	370^e^	ND	ND	ND	[43, 44]
	**38**	0.939^e^	88.3^f^	ND	ND	[44]
	**39**	0.745^e^	247^f^	ND	ND	[44]
	*A_2A_ AR antagonists*					
	**40**	647	48	ND	ND	[45]
	**41**	468	3	ND	ND	[45]
	**42**	206	1	ND	ND	[45]
	*A_3_ AR antagonists*					
	**43**	334	728.1	49.8^n^	0.60	[46]
	**44**	1,037	3,179	53.9^n^	0.18	[46]
	Pyrazolo-[1,5-*a*]pyridine					
	*A_1_ AR antagonists*					
	**45,** FK453	17^o^	11,000^p^	ND	ND	[47]
	**46**, FK838	120^o^	5900^p^	ND	ND	[48]
	**47**, FR166124	15^o^	6200^p^	ND	ND	[49]

ND: Not determined.

^a, b, c, d^: binding affinity assay determined using recombinant cells expressing human A_1_ AR, A_2A_ AR, A_2B_ AR, and A_3_ AR, respectively, unless noted.

^e^: binding affinity assay determined at A_1_ AR in rat brain membranes.

^f^: binding affinity assay determined at A_2A_ AR in rat striatal membranes.

^g^: adenylyl cyclase assay determined using recombinant cells expressing human A_2B_ AR.

^h, i, j, k^: IC_50_ value from binding affinity assay determined with human A_1_ AR, A_2A_ AR, A_2B_ AR, and A_3_ AR, respectively.

^l^: binding affinity assay determined at A_1_ AR in bovine cerebral cortical membranes.

^m^: binding affinity assay determined at A_2A_ AR in bovine striatal membranes.

^n^: IC_50_ value from adenylyl cyclase assay determined at human A_2B_ AR.

^o^: IC_50_ value from binding affinity assay determined at A_1_ AR in rat brain membranes.

^p^: IC_50_ value from binding affinity assay determined at A_2A_ AR in rat striatal membranes.

## Abbreviations

### Abbreviations

AR(s): adenosine receptor(s)
cAMP: cyclic adenosine monophosphate
Cl-IB-MECA: 2-chloro-N^6^-(3-iodobenzyl)-5′-N-methylcarboxamidoadenosine
ERK 1/2: extracellular signal-regulated kinase
8FB-PTP: 5-amino-8-(4-fluorobenzyl)-2-(2-furyl)-pyrazolo[4,3-*e*]-1,2,4-triazolo[1,5-*c*]pyrimidine
FK453: 2-phenylpyrazolo[1,5-*a*]pyridine-3-((2*R*)-2-(2-hydroxyethyl)piperidyl)acryloylamides
FK838: 3-(2-butyric acid-3-oxo-2,3-dihydropyridazin-6-yl)-2-phenylpyrazolo[1,5-*a*]pyridine
FR166124: 3-(2-cyclohexenyl acetic acid-3-oxo-2,3-dihydropyridazin-6-yl)-2-phenylpyrazolo[1,5-*a*]pyridine
GPCR: G protein-coupled receptor
[^3^H]MRE-3008-F20: [^3^H]-5-N-(4-methoxyphenylcarbamoyl)amino-8-propyl-2-(2-furyl)pyrazolo[4,3-*e*]-1,2,4-triazolo[1,5-*c*]pyrimidine
IB-MECA: N^6^-(3-iodobenzyl)-5′- N-methylcarboxamidoadenosine
MAPK: mitogen-activated protein kinase
MRS5346: 5-((2-(2-(4-(3-(5-amino-2-(furan-2-yl)-7H-pyrazolo[4,3-*e*][1, 2, 4]triazolo[1,5-*c*]pyrimidin-7-yl)propyl)phenoxy)acetamido)ethyl)-carbamoyl)-2-(6-amino-3-iminio-4,5-disulfonato-3H-xanthen-9-yl)benzoate
NECA: N-ethylcarboxamidoadenosine
PET: positron emission tomography
SCH 63390: 5-amino-7-(3-phenyl propyl)-2-(2-furyl)pyrazolo[4,3-*e*]1,2,4-triazolo[1,5-*c*]pyrimidine
SCH 58261: 5-amino-7-(*β*-phenylethyl)-2-(2-furyl)pyrazolo[4,3-*e*]1,2,4-triazolo[1,5-*c*]pyrimidine
SCH 442416: 5-amino-7-[3-(4-methoxyphenyl)propyl]-2-(2-furyl)pyrazolo[4,3-*e*]-1,2,4-triazolo[1,5-*c*]pyrimidine
SCH 420814: 5-amino-7-{[(2-(4-methoxyethoxyphenyl)piperazin-1-yl]ethyl}-2-(2-furyl)-pyrazolo [4,3-*e*]1,2,4-triazolo[1,5-*c*]pyrimidine.
